# A Rare Case of a Primary Squamous Cell Carcinoma of the Stomach Presenting as a Submucosal Mass

**DOI:** 10.1155/2015/482342

**Published:** 2015-06-21

**Authors:** Wolf von Waagner, Zhuo Wang, Antonio I. Picon

**Affiliations:** ^1^Division of Surgical Oncology, Department of Surgery, Staten Island University Hospital, 256 B Mason Avenue, Staten Island, NY 10305, USA; ^2^Department of Pathology, Staten Island University Hospital, 475 Seaview Avenue, Staten Island, NY 10305, USA

## Abstract

We report a case of a 70-year-old man, with a status after aortic valve replacement, who presented with melena and hypotension. On physical examination, he was hypotensive, but he responded to resuscitation. Esophagogastroduodenoscopy revealed a submucosal mass in the gastric fundus. Imaging of the chest, abdomen, and pelvis showed no evidence of local or distant metastasis. He underwent a partial diaphragmatic resection, gastrectomy, lymphadenectomy, and Roux-en-Y esophagojejunostomy. Pathology showed a gastric squamous cell carcinoma (SCC) invading the diaphragm, with negative margins of resection, and one positive perigastric lymph node. He received chemoradiation, but the patient expired 27 months after surgery.

## 1. Introduction

Primary gastric squamous cell carcinoma (PGSCC) is an extremely rare neoplasm. It accounts for 0.04–0.07% of all gastric carcinomas [1–5], with fewer than one hundred cases having been reported in the literature [6]. The pathogenesis of this tumor remains unclear; most of the cases have a late diagnosis. The optimal treatment strategy is controversial and the prognosis is poor. Although the incidence of gastric cancer in Japan is much higher than that in Western countries, primary gastric SCC is still rare [1, 2]. We report a case of advanced PGSCC, who presented with melena and an ulcerated submucosal mass in the fundus of the stomach. The patient underwent radical resection and received adjuvant chemoradiation therapy. The disease progressed on chemotherapy and he expired 27 months after surgery.

## 2. Case Report

**Figure 1:**
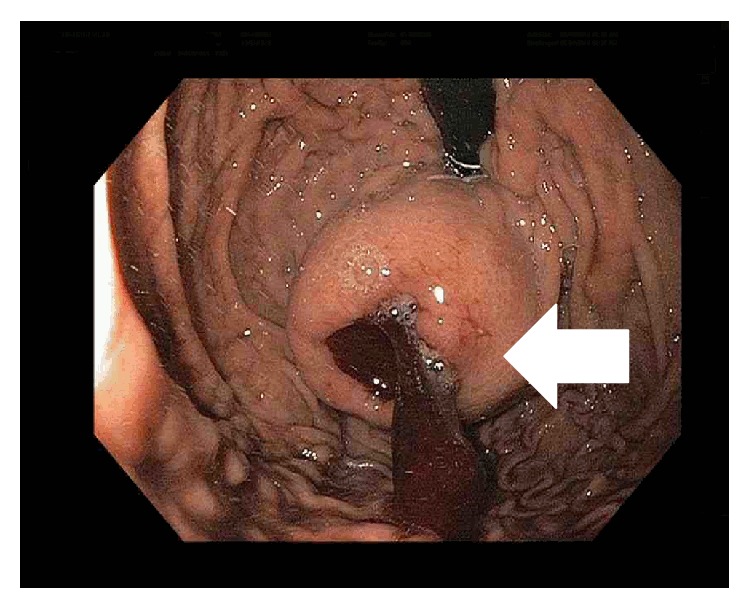
Esophagogastroduodenoscopy (EGD) showing an ulcerated submucosal mass in the fundus of the stomach (white arrow).

**Figure 2:**
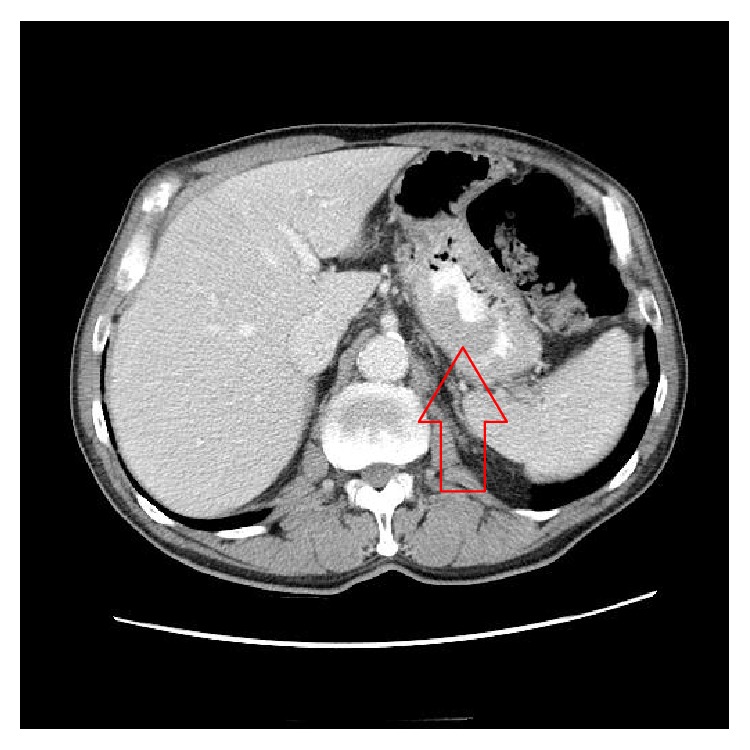
Computerized axial tomography scan with IV and PO contrast showing a mass in the gastric fundus (red arrow).

We report a case of a 70-year-old white male, with a status after uncomplicated aortic valve replacement for severe aortic stenosis, who presented with melena and hypotension, returning to the hospital the same day of discharge. He reported a 15-pound weight loss over a few months. His past medical history was significant for a 60-pack-year smoking history and severe aortic stenosis. On physical examination, he was pale, tachycardic, and hypotensive, but he responded well to resuscitation. Esophagogastroduodenoscopy (EGD) revealed a seven-centimeter ulcerated submucosal mass in the fundus of the stomach without active bleeding (Figure 1), and biopsy was not attempted. Imaging of the chest and abdomen revealed a 7 × 4 cm mass in the gastric fundus with no evidence of locoregional extension or distant metastasis (Figure 2). He was taken to the operating room and it was found that the mass was locally invading the left hemidiaphragm. He underwent a partial left diaphragmatic resection, total gastrectomy, D1A lymphadenectomy, reconstruction with Roux-en-Y esophagojejunostomy, and a feeding tube jejunostomy. Histological and immunohistochemical analysis revealed an infiltrating moderately differentiated gastric squamous cell carcinoma (SCC) with direct invasion to the adjacent diaphragm striated muscle, with free margin resection, and one perigastric lymph node was positive for metastatic disease for a T4, N1, and M0 disease. Immunohistochemistry was positive for cytokeratin 5/6, p63 and negative for CD117, CK20, and p16. Three months after surgery, he was started on adjuvant radiation therapy and chemotherapy with capecitabine and oxaliplatin. He developed recurrent disease in the peritoneum and multiple liver metastases were found on positron emission tomography scan (PET). He received sorafenib, but he presented severe fatigue and the dose was decreased and eventually stopped. Carboplatin plus irinotecan was started 16 months after surgery due to progression of liver metastases. Twenty months after surgery, imaging showed progression of disease and 5-fluorouracil and gemcitabine were started with no response. Subsequently, he had progression of disease and expired 27 months after surgery.

## 3. Pathology 

**Figure 3:**
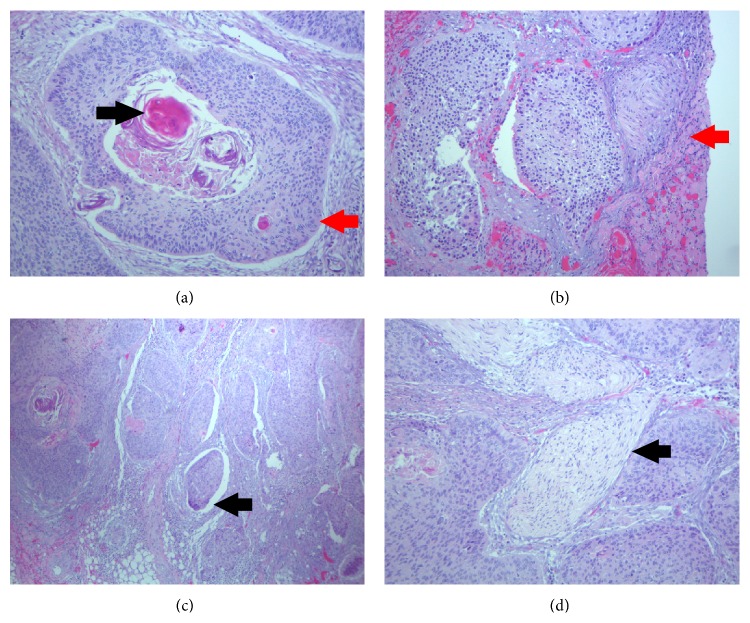
(a) Histopathological examination showing a moderately differentiated squamous cell carcinoma with keratinization (×100). Keratin pearl (black arrow). Mosaic cell arrangement with sharp border (red arrow). (b) Tumor invasion of the diaphragm (×100) (red arrow). (c) Lymphovascular invasion is present (×40) (black arrow). (d) Tumor with nerve invasion (×40) (black arrow).

**Figure 4:**
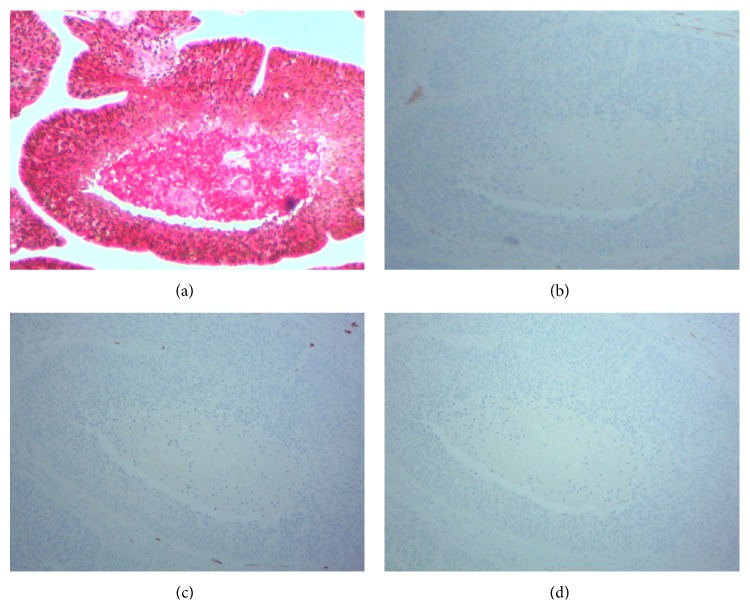
Tumor cells showing strong coexpression of p63 (nuclear stain, brown) and cytokeratin 5/6 (membrane stain, red) (a) and negative expression of p16 (b), CD117 (c), and CK7 (d).

The surgical specimen was composed of a total gastrectomy with partial resection of adherent diaphragm. There was a submucosal tan/white soft mass measuring 4.0 × 3.5 × 3.5 cm, with focal necrosis, located at the fundus. The tumor did not extend to the surgical specimen margins. Examination of the gastric and duodenal tissue revealed no further tumor mass. Histologically, the tumor showed moderately differentiated squamous cells with keratinization and without glandular differentiation (Figure 3(a)). The tumor located predominantly in the submucosa through the serosa and with direct invasion to the adjacent diaphragm striated muscle (Figure 3(b)). Lymphovascular invasion and perineural invasion were observed adjacent to the tumor (Figures 3(c) and 3(d)), respectively. Further immunohistochemistry showed tumor cells with strong coexpression of CK5/6 and p63, which are indicators of squamous cell carcinoma (Figure 4(a),* dual staining*), but negative for p16 (Figure 4(b)), CD117 (indicator of gastrointestinal stromal tumor, Figure 4(c)), and CK7 (indicator of adenocarcinoma, Figure 4(d)). One perigastric lymph node (1/13) exhibited metastatic squamous cell carcinoma.

## 4. Discussion 

The primary gastric squamous cell carcinoma (PGSCC) has a worldwide incidence that is estimated to be about 0.04 to 0.07% among other gastric cancers [1–5]. It occurs mostly in men and the male/female ratio is 5 to 1 [1, 7, 8]. It is more prevalent in the sixth decade of life [5], and the most common tumor location is in the upper third of the stomach [1]. The gender, age at the time of presentation, and location of our patient fit the epidemiology of this unusual neoplasm. Rörig, who described the first PGSCC case in 1895, hypothesized about basal cells in the gastric mucosa undergoing metaplasia, transforming into squamous cells, and later turning into SCC [9]. Since then, not much has changed; the etiopathogenesis is still unknown, although several theories have been proposed [5, 9, 10]. Five theories were summarized by Straus et al. [4, 5]: (i) totipotential stem cells in the gastric mucosa [11]; (ii) nest of ectopic squamous epithelium in the gastric mucosa [5, 12]; (iii) squamous metaplasia of preexisting nonneoplastic glandular epithelium where metaplasia is induced by noxious agents as gastric acid in the mucosa surrounding peptic ulcers [3, 7, 12], corrosive acid ingestion [13], gastric tuberculosis [14], and so forth; (iv) squamous differentiation in a preexisting adenocarcinoma, supported by a report of three PGSCC tumors which were reexamined and areas of adenocarcinoma were found. The author suggests that stem cells turn into adenocarcinoma, and later squamous metaplasia occurs and finally SCC appears [7, 15]. (v) Other authors proposed origin from endothelium of gastric vessels [16, 17]. However, it was deemed a very unlikely origin, due to the absence of specific vascular endothelium markers in squamous cells [6]. We particularly stand for the squamous metaplasia of a normal gastric mucosa, because this hypothesis is backed up by reports of PGSCC tumors thought to have pure SCC cells and later showed to have glandular components [4, 7, 15, 18].

The histopathological criteria for PGSCC were described by Boswell and Helwig in 1965 [5, 7, 12], and at least one of the following must be present to make the diagnosis: (A) keratinized cell masses forming keratin pearls, (B) a mosaic cell arrangement, in which cell borders are sharp, (C) intercellular bridges, and (D) high concentrations of sulphydryl or disulphide bonds, indicating the presence of keratin or prekeratin sulphydryl or disulphide bonds. We found two, the keratin pearls and the mosaic cell arrangement with sharp border. The intercellular bridges are usually present in the well differentiated squamous cell carcinoma. In this case, however, these particular features were not seen, which indicates that cells lost tight-junction or cell-cell interaction, with the latter being a feature of poorly differentiated tumors.

Parks published in 1967 three diagnostic criteria for PGSCC, designed to exclude esophageal tumors and other primary sources [5, 7, 15, 19, 20], absence of tumor in the cardia, noninvolvement of esophagus, and no other primary SCC elsewhere.

In our case, there was absence of tumor in the cardia, noninvolvement of the esophagus, and the presence of other tumors in the patient that was ruled out by further imaging. We could label this tumor as primary gastric squamous cell carcinoma.

Another classification is the diagnostic criteria for primary SCC of the stomach, by the Japanese Classification of Gastric Carcinoma [21]. All tumor cells are SCC cells, with no adenocarcinomatous components in any sections, and there is distinct evidence that SCC arises directly from the gastric mucosa [1, 21]. This tumor also meets these criteria, despite the differences with the Japanese literature, which do not exclude the cardia.

Some immunohistochemistry studies have found the dual expression of both p63 and cytokeratin 5/6 with a specificity of 99% and a sensitivity of 98% for squamous cell carcinoma [22]. In this case, the tumor cells had positive immunoreactivity for p63 and CK5/6, supporting the diagnosis of PGSCC.

Although chemotherapy combined with surgical resection is reported to improve survival [3, 7], currently, there is no consensus on how to treat PGSCC. Different chemotherapeutic regimes have been used, but there is no standard course recommended in the literature [3, 5, 20]. Our patient received adjuvant radiation therapy and chemotherapy with capecitabine and oxaliplatin. He had a disease-free interval of ten months, but eventually he developed tumor recurrence in the liver and peritoneum.

Radical surgical resection is the treatment of choice when there is no evidence of disease outside the stomach. Radical resection for locally advanced tumors to obtain negative margins has been suggested [5]. Our surgical procedure was a total gastrectomy with a D1A lymphadenectomy, one perigastric lymph node out of 13 was positive, which was adjacent to the mass, “en bloc” resection with part of the left diaphragm was necessary, and the reconstruction was done with a Roux-en-Y esophagojejunostomy.

In conclusion, we have documented a PGSCC that meets the current diagnostic criteria; this tumor is so uncommon that there are no data to support radical resection, lymphadenectomy, and chemoradiation. In view of the fact that this particular tumor tends to metastasize to lymph nodes and is locally aggressive and the recurrence rate (local and distant metastases) is high, we recommend aggressive local therapy and adjuvant chemoradiation therapy.
